# Study of β-catenin, E-cadherin and vimentin in oral squamous cell carcinoma with and without lymph node metastases

**DOI:** 10.1186/1746-1596-9-145

**Published:** 2014-07-21

**Authors:** Partheeban Balasundaram, Manoj Kumar Singh, Amit Kumar Dinda, Alok Thakar, Rajni Yadav

**Affiliations:** 1 Department of Pathology, All India Institute of Medical Sciences, New Delhi, India; 2 Department of Otorhinolaryngology and Head & Neck Surgery, All India Institute of Medical Sciences, New Delhi, India

**Keywords:** Squamous cell carcinoma, Metastases, Lymph node, β-catenin, Vimentin, E-cadherin

## Abstract

**Abstract:**

**Background**

Despite great improvement in the surgical treatment and adjunctive therapy for oral squamous cell carcinoma (OSCC), prognosis remains dismal in advanced cases. Regional metastatic disease is known to reduce recurrence free survival and disease specific survival significantly. The present study was conducted to evaluate the role of cell adhesion molecules β-catenin, E-cadherin and vimentin in predicting tumour metastasis of OSCC.

**Methods**

A total of sixty cases of oral squamous cell carcinoma were included for the study which comprised of 30 cases with lymph node metastases and 30 cases without metastases. Immunohistochemistry was performed for β-catenin, E-cadherin and vimentin on both the test groups along with 30 controls from normal buccal mucosa and inflammatory lesions each.

**Results**

There was no significant difference between the immunoreactivity for β-catenin, E-cadherin and vimentin between OSCC with and without lymph node metastases. Vimentin immunopositivity was noted with varying intensity in all cases of OSCC.

**Conclusions**

E-cadherin and β-catenin are probably not the key determinants for regional metastases in OSCC. The role of vimentin expression in OSCC and metastases is controversial and needs to be studied further.

**Virtual slides:**

The virtual slide(s) for this article can be found here: http://www.diagnosticpathology.diagnomx.eu/vs/6506095201182002.

## Background

Oral squamous cell carcinoma (OSCC) is the sixth most common malignancy in the world and ranks as first in males in the Indian subcontinent. It is a major cause of cancer morbidity and mortality [1]. Despite great improvement in surgical treatment and adjunctive therapy, prognosis remains dismal in advanced cases. Regional metastatic disease is known to reduce recurrence free survival and disease specific survival significantly [2].

Adhesion molecules play a central role in pathogenesis and progression of malignant tumours [3]. Vimentin is an intermediate filament found predominantly in mesenchymal cells, but not in epithelial cells. However, it also exists in some carcinoma cell lines [4-6] and squamous cell carcinomas [7,8].

Therefore, it is important to evaluate the role of cell adhesion molecules like β-catenin and E-cadherin along with vimentin in tumour metastasis of OSCC. In this study, we studied the immunohistochemical expression of vimentin, β-catenin and E-cadherin in oral squamous cell carcinoma with and without lymph node metastasis.

## Methods

### Patients and tissue specimens

A total of 60 cases of primary OSCC diagnosed over a period of 2 years (2010–2012) in the Department of Pathology, All India Institute of Medical Sciences were included for the study. All the patients had been surgically treated with tumour resection and radical neck dissection. None of the patients did receive any tumour specific therapy (chemotherapy or radiotherapy) before the resection. Thirty cases with a clinical suspicion of malignancy but diagnosed as inflammatory lesions on histology and 30 histologically confirmed normal mucosal margins from the resection specimens were included as control group. This study was approved by the Ethics Committee of All India Institute of Medical Sciences.

### Histopathological evaluation

Specimens from all the cases were fixed in 10% formaldehyde solution and embedded in paraffin. Histological diagnosis was made on haematoxylin and eosin stained sections according to the revised criteria given by the World Health Organization (2005). OSCCs were classified into well, moderately and poorly differentiated grades. Staging was done based on TNM staging into four categories, stage I-IV.

### Immunohistochemistry

Tissue sections (4 μm) cut from representative paraffin blocks were deparaffinised in xylene and rehydrated through graded alcohols. Endogenous peroxidase was blocked using 4% hydrogen peroxide. For antigen retrieval, the sections were processed by conventional microwave heating in 10mMol/L sodium citrate retrieval buffer (pH 6.0) for 30 minutes. The sections were then incubated for overnight with primary antibody at 4°C in a humid chamber for β catenin (Spring bioscience), E-cadherin (Spring bioscience) and vimentin (Thermo scientific). The dilutions used for the primary antibodies were 1:250, 1:100 and 1:400 for β catenin, E-cadherin and vimentin respectively. The sections were subsequently incubated with anti-mouse immunoglobulin in phosphate buffered saline (PBS) containing carrier protein and 15 mM Sodium Azide (large volume universal DAKO LSAB kit, Peroxidase, M/s Dakopatts, Denmark) at room temperature for 30 minutes for β-catenin, E-cadherin and vimentin. The sections were then washed three times with PBS (pH 7.2) for 2 min. The reaction product was developed with 3, 30-diaminobenzidine and counterstained with haematoxylin. Immunoreactivity in the tissue was judged independently by two pathologists who were blinded to the clinical data and other immunohistochemical results. Normal oral mucosal tissues were used as positive control. Negative controls were included in each slide run with omission of primary and secondary antibodies.

### Evaluation of immunoreactivity

Immunoreactivity was semi quantitatively evaluated on the basis of staining intensity and distribution using the immunoreactive score [9,10].

Immunoreactive score = intensity score x proportion score. The intensity score was defined as 0: negative; 1: weak; 2: moderate; or 3: strong, and the proportion score was defined as 0: negative; 1: <10%; 2: 10-50%; 3: >50-80%; or 4: >80% positive cells. The total score ranged from 0 to 12. Immunoreactivity was divided into three groups based on the final score: negative immunoreactivity was defined as a total score of 0, low immunoreactivity was defined as a total score of 1–4, and high immunoreactivity was defined as a total score >4.

### Statistical analysis

The correlation between clinicopathological parameters and β catenin, E-cadherin and vimentin expression were analysed using the chi square test and Fisher’s exact test. A p value <0.05 was considered statistically significant.

## Results

### Clinicopathological characteristics

A total of 60 cases of OSCC were analyzed, of which 30 cases had lymph node metastasis. Majority of them were males (77%) and females constituted 14 (23%) cases. Age range was 23 years to 72 years with a mean age of 44.79 years. Tongue was the most common site involved (52%). Well differentiated, moderately differentiated and poorly differentiated squamous cell carcinomas included 38, 20 and 2 cases respectively. Of 60 cases, 21(35%) cases were Stage III, 17(28%) Stage IV, 16(27%) Stage I and 6(10%) in Stage II.

### Immunohistochemical analysis

**Figure 1:**
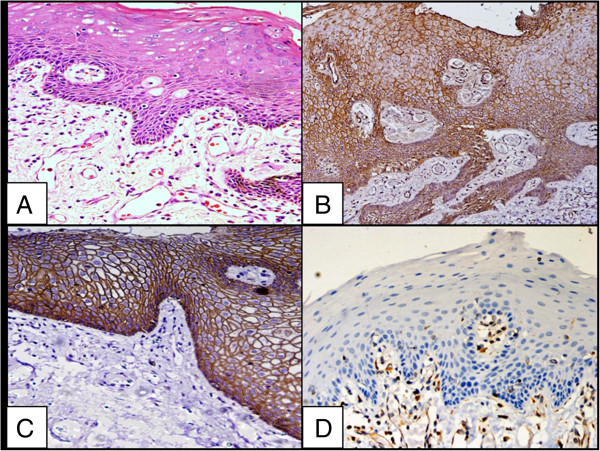
**Staining pattern of normal buccal mucosa. A**. Normal buccal mucosa, H&E X200; **B**. High immunoreactivity of β-catenin in normal buccal mucosa X200; **C**. High immunoreactivity of E-cadherin in normal buccal mucosa X200; **D**. Absence of immunostaining for vimentin in normal squamous epithelium of buccal mucosa X200.

**Figure 2:**
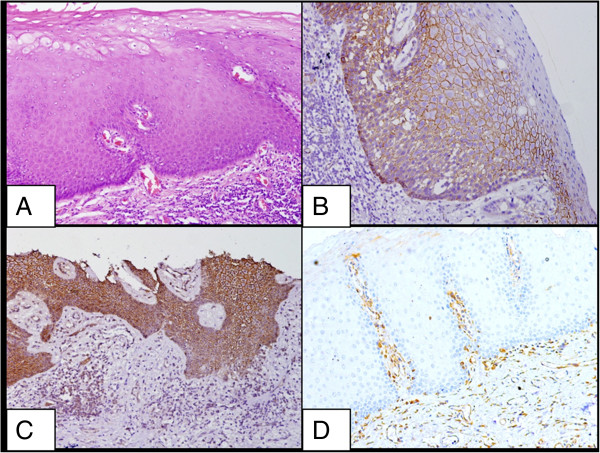
**Staining pattern of oral lichen planus. A**. Oral lichen planus, H&E XX200; **B**. High immunoreactivity for β-catenin in squamous epithelium of a case of lichen planus X200; **C**. High immunoreactivity for E-cadherin in squamous epithelium of a case of lichen planus X200; **D**. Absence of immunostaining for vimentin in squamous epithelium in a case of lichen planus X200.

Both the control groups revealed a strong membranous staining pattern of E-cadherin with an absence or reduced expression in the superficial layer of mature well differentiated cells. Strong membranous β-catenin expression was observed in the suprabasal to the basal layers with absence of staining in the superficial layer in both the control groups. Vimentin expression was detected in the cytoplasm of the connective tissue mesenchymal cells of normal oral mucosal tissue, but not in the squamous epithelium (Figures 1 and 2).

### Expression of β-catenin, E-cadherin and vimentin in OSCC patients

**Figure 3:**
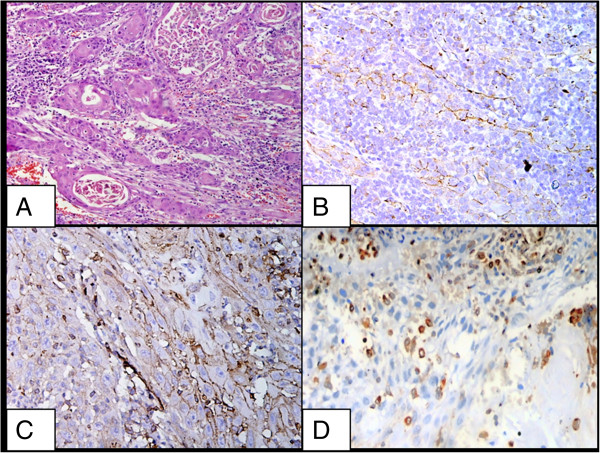
**Staining pattern of squamous cell carcinoma. A**. Squamous cell carcinoma, buccal mucosa, H&E X200; **B**. Low immunoreactivity of β-catenin in a case of squamous cell ]carcinoma X200; **C**. Low immunoreactivity of E-cadherin in a case of squamous cell carcinoma X200; **D**. Low immunoreactivity for vimentin in a case of squamous cell carcinoma X200.

Comparison of expression of β-catenin, E-cadherin and vimentin in OSCC with and without lymph node metastases is enumerated in Table 1. OSCCs showed a weaker expression of both β-catenin and E-cadherin than the control groups (p <0.05). However, there was no significant difference in the degree of β-catenin (p = 1.000) and E-cadherin (p = 0.771) expression in study groups of OSCC with and without lymph node metastases. Vimentin expression was seen in all cases of OSCC. Vimentin was expressed in the cytoplasm of the tumour cells with weak to moderate intensity. However, there was no significant difference in the degree of vimentin (p = 0.360) expression in both the study groups (Figure 3).

**Table 1:** Comparison of OSCC with and without lymph node metastases

**Groups**	**Beta catenin**		***p*** **value**	**E- cadherin**		***p*** **value**	**Vimentin**		***p*** **value**
	**≤4**	**5–12**		**≤4**	**5–12**		**≤4**	**5–12**	
OSCC without lymph node metastasis (n = 30)	9	21	0.771	7	23	1.000	25	5	0.360
OSCC with lymph node metastases (n = 30)	7	23		6	24		21	9	

Patients without lymph node metastases were nearly equally distributed in age groups of ≤50 years and >50 years where as patients with lymph node metastases predominantly belonged to the ≤50 years group. There were no statistically significant differences between the clinical variables (age, sex, site, size of tumour and histological differentiation) in OSCC with and without lymph node metastases as enumerated in Table 2.

**Table 2:** Correlation of clinical variables in OSCC with and without lymph node metastases

**Variable**	**OSCC without lymph node metastases (n = 30)**	**OSCC with lymph node metastases (n = 30)**	***P*** **value**
Age: ≤ 50 yrs	16	24	0.054
> 50 yrs	14	6	
Sex: Female	6	8	0.761
Male	24	22	
Site: Tongue	14	17	0.605
Buccal mucosa	16	13	
Size: ≤ 4 cm	24	23	1.000
> 4 cm	6	7	
Differentiation: WDSCC	17	21	0.698
MDSCC	12	8	
PDSCC	1	1	

There was no statistically significant difference between the expressions of β-catenin, E-cadherin or vimentin and clinicopathological variables (age, sex, site, size of tumour, histological differentiation and stage) in OSCCs with and without lymph node metastases as listed in Table 3. Thus, all these variables did not affect the expression of adhesion molecules.

**Table 3:** Correlation of β catenin, E-cadherin and vimentin expression level of tumours and clinical variables

**Variable**	**Total cases(n = 60)**	**β catenin**		***P*** **value**	**E-cadherin**		***P*** **value**	**Vimentin**		***P*** **value**
		**Low**	**High**		**Low**	**High**		**Low**	**High**	
Male	46	15	31	0.085	10	36	1.000	34	12	0.484
Female	14	1	13		3	11		12	2	
≤ 50 yrs	40	11	29	1.000	12	28	0.768	29	11	0.347
> 50 yrs	20	5	15		5	15		17	3	
Tongue	31	8	23	1.000	8	23	0.535	25	6	0.547
Buccal mucosa	29	8	21		5	24		21	8	
WDSCC	38	10	28	0.538	6	32	0.390	31	7	0.200
MDSCC	20	4	16		6	14		13	7	
PDSCC	2	1	1		0	2		1	1	
≤ 4 cm	47	12	35	0.731	10	37	1.000	35	12	0.713
> 4 cm	13	4	9		3	10		11	2	
Stage I&II	22	6	16	1.000	3	19	0.338	18	4	0.542
Stage III&IV	38	10	28		10	28		28	10	

## Discussion

Oda T et al. proposed three mechanisms of alteration of cadherin-mediated cell adhesion system in human cancers in vivo and in vitro. The first is down-regulation of E-cadherin expression and its gene mutation [11]. Existence of altered E-cadherin expression in human cancers and a significant relationship between reduced E-cadherin expression and clinicopathological factors, such as dedifferentiation, invasiveness, or metastasis was previously reported [12]. The second is abnormality or deletion of catenins [13,14]. The third abnormality is biochemical modification of catenins. It has been demonstrated that tyrosine phosphorylation of β-catenin induced by various factors (eg. v-src, hepatocyte growth factor and epidermal growth factor) suppresses E-cadherin function in vitro [15].

The present study revealed the down regulation of molecular markers β-catenin and E-cadherin in OSCC along with aberrant expression of vimentin. However, the present study did not show any significant difference in the expression of E- cadherin, β- catenin and vimentin in OSCC with and without lymph node metastases.

As proposed by various studies [16-19], there was decreased expression of E-cadherin and β-catenin in OSCC tumour cells, in comparison to strong expression in the control groups. This was statistically significant in OSCC without lymph node metastasis (p = 0.002, p = 0.011) and OSCC with lymph node metastases (p = 0.011, p = 0.024).

Epithelial-mesenchymal transition is an important biological process during development and oncogenesis. It is characterized by a reduction of epithelial polarities and production of mesenchymal phenotypes. Down regulation of epithelial marker like E-cadherin and increase in mesenchymal marker vimentin are hallmarks of transition [20,21].

Few studies have detected the expression of vimentin in OSCC patients and cell lines [22,23]. Similar feature was noted in the present study.

Down regulation and loss of E-cadherin was associated with lymph node metastases and advanced stage of OSCC according to various studies [18,24-26] while few studies [16,17,27,28] failed to prove this prognostic value. The present study followed the latter trend.

Reduced staining intensity of the E-cadherin molecule has been correlated by Rodrigo et al. with the presence of nodal metastatic disease in a study on 101 patients with supraglottic laryngeal carcinoma [29] although, few studies failed to show this relationship [27,30]. Bukholm et al. [31] reported that there was no significant difference between the expression of E-cadherin and the presence of regional metastasis in human breast cancer, and it is said that the significance of changes in the E-cadherin complex may vary from tumour to tumour [32].

Reduced β-catenin staining was a predictive marker for lymph node metastases in OSCC according to few studies [24,33,34] and not by others [16,27,35]. The present study did not have a significant difference in the reduced expression of β-catenin and lymph node metastases in OSCC.

Several factors related to methodology may account for the discrepancy in results. The method of evaluation of immunostaining and the definition of under-expression is quite variable, hence compromising accurate comparison of data.

In some studies, a semi-quantitative estimation of the immunoreactive intensity was used [24,25,33,36,37] with varying percentage criteria, while other studies have not assessed expression according to the percentage of positively stained cells [38,39]. Moreover, the expression analysis has been carried out in different areas of the tumour by different investigators. Some studies have focussed on the invasive tumour front [33,38] while other studies have examined expression in random areas [24,36,37,39] within the tumour. It would, however, appear from the results in the present study and other studies on OSCC [16,17,25,27,28,35,36,39] that E-cadherin and β-catenin are probably not the key determinants for regional metastases in OSCC.

In this study, all tumors in both the groups expressed cytoplasmic positivity for vimentin with varying intensity. Of 60 cases of OSCC, 45 cases (75%) exhibited a low immunoreactive score and 15 cases (25%) exhibited high immunoreactive score. Poorly differentiated OSCC constituted two cases, of which one exhibited high immunoreactive score (50%). The number of cases in the poorly differentiated carcinoma category was less for a valid comparison.

Various molecules have been studied in head and neck carcinomas. Low level of p27 expression has been reported an unfavorable prognostic factor for patients with nasopharyngeal carcinoma [40]. CypA/MMP9 signal pathway and up-regulation of USP9X may be attributed to the malignant transformation of esophageal squamous cell carcinoma (ESCC) [41,42]. Also, over-expression of ABCG2 and V-ATPase is noted in ESCC. RNAi targeting CXCR4 is known to inhibit proliferation and invasion of esophageal carcinoma cells [43]. Both ABCG2 and V-ATPase are over-expressed in esophageal squamous cancer cells [44]. Some new molecules are being studied in head and neck squamous cell carcinomas (HNSCC). Mir-205 has been demonstrated as a novel molecular marker for the detection of metastatic HNSCC [45]. In another study, primary tumors and positive nodes of the metastatic cases revealed decreased expression of collagen XVIII and CBP2/HSP47 in metastases [46]. More studies on role of integrins, epidermal growth factor receptor, matrix metalloproteinases, cathepsins, chemokine receptors and angiogenesis markers are needed for future.

## Conclusions

Expression of β-catenin and E-cadherin is though downregulated in oral squamous cell carcinomas, it solely does not predicts lymph node metastasis. Aberrant vimentin expression is seen in these tumours. Loss of intercellular adhesion is only one of the stages required for the occurrence of metastases; there could be a need for other phenomena, such as loss of cell adhesion to the extracellular matrix, which can be caused by the metalloproteinases expression, which has to be further evaluated. Detailed studies on KCl co transporter −3 (KCC 3) functions with emphasis on disruption of E-cadherin/β–catenin complex formation and cell-cell junction dysfunction are required.

## Abbreviations

OSCC: Oral squamous cell carcinoma.

## Competing interests

No financial/funding sources. The authors declare that they have no competing interests.

## Authors’ contributions

PB contributed in designing and execution of the study. MKS participation in the conception and critical revision of the study. AKD participated in the interpretation of data and intellectual revision. AT made contribution to acquisition of clinical data. RY participated in analysis of data and drafting the manuscript. All authors have read and approved the manuscript.
